# Public health, pluralism, and the telos of political virtue

**DOI:** 10.1007/s40592-024-00216-z

**Published:** 2024-10-03

**Authors:** Kathryn L. MacKay

**Affiliations:** Sydney Health Ethics, University of Sydney, Camperdown, NSW 2006 Australia

**Keywords:** Public health, Virtue ethics, Justice, Teleology, Non-oppression

## Abstract

In the ethics of public health, questions of virtue, that is, of what it means for public health to act excellently, have received little attention. This omission needs remedy first because achieving improvements in population-wide health can be in tension with goals like respect for the liberty, self-determination, or non-oppression of various individuals or groups. A virtue-ethics approach is flexible and well-suited for the kind of deliberation required to resolve or mitigate such tension. Public health requires practically wise and careful thinking, which virtue ethics brings with it. Furthermore, too tight a focus on delivering outcomes in determining how public health should act has, in some cases, actually undermined its ability to achieve those consequences. However, the main concern about incorporating virtue into public health in a pluralistic society is likely to be that virtue is generally teleological, and we would surely need some widely agreed upon idea of something like flourishing or the common good for this to work. In this paper, I propose that for public health to express virtue in its work, it must express a commitment to justice as it goes about its business promoting and protecting the health of the population. Justice is both a contributor toward better health for groups in society, and a worthwhile goal in its own right. I will sketch an argument that justice as non-oppression – not merely health equity – is the right telos toward which excellent public health should aim in a pluralist society.

The raison d’etre of public health is to improve health and well-being at the level of the entire community or population. Making such improvements, whether related to chronic conditions or infectious diseases, requires the collective to act in the way that public health wants it to act, and this requires a degree of cooperation. To get such cooperation in a democratic society as highly diverse as Australia, Canada, or the United States, public health has to be acting in a way that is acceptable to most people, making the ethics of public health a highly political area.

In the ethics of public health, questions of virtue, that is, questions of what it means for public health to act excellently, have received little attention. There are a few reasons why this omission needs remedy. One reason is that delivering public health outcomes can be in tension with goals like respect for the self-determination or non-oppression of different groups, or respecting liberty. A virtue-ethics approach is flexible and well-suited for the kind of deliberation required to resolve or mitigate such tension. Public health requires practically wise and careful thinking, which virtue ethics brings with it. Furthermore, too tight a focus on delivering outcomes in determining how public health should act has, in some cases, actually undermined its ability to achieve those consequences. Some of the activities public health undertakes and justifies by appeal to better health outcomes can be positively unkind, disrespectful, or unfriendly toward various groups of people.

However, capturing that something is unkind or disrespectful in the typical language of public health ethics, even deontic language around liberty or autonomy, is difficult. Public health ethics has not had a way to analyse the motivations behind practices or interventions, or to advise how these could be made better. It has had limited ways in which to articulate what it means for public health to act well. This is a problem because the ends for which public health acts and the means by which it achieves them need careful consideration, beyond immediate outcomes, and this requires the excellence of practical wisdom. Virtue ethics brings with it concepts of excellent deliberation and discernment, moral motivation, right action, and moral improvement, and would make public health practice more ethically robust. However, the main concern about incorporating virtue into public health in a pluralistic society is likely to be that virtue is generally teleological, and we would surely need some widely agreed upon idea of something like flourishing or the common good – both of which concepts are energetically debated – in order for this to work.

I have a more modest proposal for the telos of public health than settling on an idea of flourishing. In the context of the political, justice is what is good. This is different from the individual good, as it must be. For public health to express virtue in its work, it must express a commitment to justice as it goes about its business promoting and protecting the health and wellbeing of society. Justice is both a contributor toward better health for groups in society, and a worthwhile goal in its own right. So, though I do not have space to flesh out a full account, I will sketch an argument that justice as non-oppression – not merely health equity – is the right telos toward which public health should aim in a pluralist society.

## Virtues in institutions

I will begin with a brief explanation of what I think it means for institutions to express virtue. In my account of political virtues for institutions like public health, I utilise Aristotle’s account of personal virtues to draw an analogy with political virtues. Aristotle argued that virtues were delineated by looking at their spheres of action. This is a helpful way to discover the virtues relevant to public health, and other social institutions. By considering the spheres of public health action, we can determine what it means for public health to be excellent or fine. Political virtues, once delineated, are characteristic dispositions of institutions, which are beneficial to society and corrective to vicious states of affairs, and excellences of institutional agency, where excellence is understood as expressive of a collective commitment to goodness.

When I describe public health as a social institution, I mean that it is an organised system of laws, policies, practices, regulations, and customs (that is, structures) directed toward some defined purpose or aim. Structures have been usefully described by Oliver Williamson (2000) in economics, and MacIntyre (2007) in ethics as the visible and invisible influences on social behaviour, that include things as varied as norms, traditions, religious belief, constitutions, laws, policies, practices, plans, and strategies. Institutions, then, are functional groupings of structures. Examples of institutions include the banking system, education, and healthcare, as well as the market, all aspects of housing, democracy, and the various branches of a government. Institutions also maintain and create new structures through the laws, policies, rules, or strategies they introduce or uphold. In the case of public health, the purpose of this institution is typically described as promoting population-level health by reducing illness and injury, and improving health equity, and it pursues these ends through the policies, practices, campaigns, laws, or rules which it creates and maintains.

Public health is also an agent. Here, I’m employing Carole Rovane’s account, on which ‘agency’ is the result of deliberative action that achieves rational unity (Rovane 2004, 2014, 2019). On Rovane’s view, the conditions for agency are the same whether we are discussing the actions of one or many humans. So, she argues that agents can be made up of less than or more than one human life (Rovane 2019). In the case of agents like public health, different human lives come, through rational unification, to a single deliberative point of view, and so act like an *individual* agent that is larger than human size, just as an agent of human size would do. So, the agency of an institution like public health is best understood as an instance of individual agency realised at the level of a group of people.

This is an important point, for two reasons. The first reason is that in order to be held morally responsible for some act (whether praiseworthy or blameworthy), one must be understood to have agency over that act. That is, to be responsible one must have relevant capacities to be considered answerable, attributable, and accountable (Watson 1996; Shoemaker 2011). Something like a tiger, who acts on instinct, or a computer, that acts according to programming, does not meet all three of these conditions. However, an institution can have actions attributed to it (like the introduction of a new campaign), be answerable for its actions (including the justification for including certain imagery or slogans in the campaign), and held accountable for the acts and possible sequelae (such as negative impact or reception). The second reason that it is important to give an account of the agency of public health is that the public does, in fact, treat public health (and other social institutions) as if they have this agency, and in a descriptive sense, it captures what we can observe public health doing in the world. Public health *does* make change; it does create new campaigns or policies which affect different groups. So, it is important to note that we attribute agency to public health, and moreover, we are justified in doing so because it meets the criteria for agency. So, when we want to hold public health responsible for doing something, we are making a sensible claim. It is an agent, and it can be praised or blamed.

Returning to virtue, now, when considering an agent made up of one human, personal virtues are typically considered to be excellences of character. When considering an agent made up of many humans, like an institution, we are no longer considering character as such; rather, what I call ‘political’ virtues are expressed through excellent structures. Agency, as deliberative action achieving rational unity, is itself morally ambivalent. In order for agency to be excellent it must express a commitment to the good (Annas 2011, 102). For institutional agency to be excellently expressed, then, the institution’s structures must display a commitment to goodness. This means that institutions must reflectively and purposefully put in place the kinds of structures (policies, practices, rules) that promote and support fine and worthy states of affairs, and correct for or prevent certain kinds of viciousness. So, political virtue is purposeful, and does not include accidentally achieving a good way of developing, or accidentally – in the case of structures – contributing to a more virtuous society. Furthermore, what it means for political virtues to express a commitment to goodness means, I will argue below, that they aim at justice. Excellence is, as in personal virtues, a condition on both the goals of action and the means chosen to achieve them. While the individual life is lived towards the end of *eudaimonia*, I propose that justice provides the appropriate end (*telos*) for public health in pluralist societies. When I make this claim about justice, I mean it in more than a distributive sense. Indeed, justice as a virtue and the *telos* of public health means something much more substantive than typical liberal accounts. Yet, I think that a substantive account can be given that is still amenable to pluralism, and I present it below. So, to summarise so far, institutional structures are virtuous (excellences of collective agency) when they express a commitment to goodness, interpreted as creating a just society.

One final note: because a wide range of actors can be involved in public health projects including charities and NGOs, for the purposes of scope, I will limit this discussion to public health agencies that are public bureaus in a democratic welfare state. Public health agencies of this kind are government agencies, established to provide certain health-related goods for a whole society (such as clean air and water, food hygiene, or herd immunity), and which goods society thinks are important. In a pluralist society such as Australia, Canada, or the United States, there is much debate about the proper tasks of public health, and which kinds of health-related issues are appropriate for state intervention. While acknowledging this challenge to the legitimacy of public health action, I must set it aside.

## Justice as non-oppression and the ends of public health

Justice is the primary good that a political system can produce, a thought that goes back at least as far as Plato’s *Republic* and Aristotle’s *Politics* and *Nicomachean Ethics*. What this means is that if our socio-political institutions are set up well, they will produce a state of justice between people and groups. What exactly it means for justice to be a virtue of political organisation is somewhat opaque, however. According to Aquinas, the virtue of justice is ordered toward an objective standard of right as its proper object, drawn from a notion of the baseline respect that every human is due (Aquinas 1516). The source of the baseline of respect is variously posed, but I endorse a version of this claim. That is, all humans, including those with intellectual disability or severe dementia, or other things we might think affect personhood, are due equal basic respect and care from our political institutions, as members of the human community. The idea that all members of the human community are due equal basic respect provides an objective standard of right, toward which justice aims.

Further, justice aims at what is fair or equal *between* people (MacIntyre 1988, p. 119), and provides normative standards of equality, of fairness, and of impartiality. So, while justice is ordered toward an objective standard of rightness (basic respect), it is also concerned with terms of relationship. Aquinas writes “the distinctive form of human goodness exemplified by the virtue of justice is characteristically expressed through a regard for the right as instantiated in particular human interactions” (Porter 2016, p. 17). Of all the virtues, Aquinas, Aristotle, and Plato agree that justice is the one particularly concerned with relations between people (Aquinas 1516; Q57-58; Aristotle 1999). Indeed, as justice is ordered to an objective standard, which is the basic respect due all to all people, this standard provides an index against which the justness of relations between groups or individuals may be measured. So, as explained by Jean Porter, the virtue of justice is expressed by “a stable disposition to care about and to pursue just relations, informed by some sense of the point or the worth of just actions and the kinds of relationships that they generate” (Porter 2016, p. 7).

While justice has an objective ordering, which protects against relativism, it is also contextually sensitive, so that justice can be achieved in different ways and in different places and times. For Aquinas, “the norms of fairness and non-maleficence correlated with justice are relatively fixed, but they also tend to be formulated in such a way as to take account of the complex normative claims that arise in any community” (Porter 2016, p. 42). So, the community itself and the kinds and character of relations within it inform the virtue of justice by helping to give shape to what expressions of ‘fairness’, or ‘respect’, for example, might look like. MacIntyre further comments that “justice, both as a virtue of the individual and as an ordering of social life, is only to be achieved within the concrete institutionalised forms of some particular ‘*polis’*” (1988, 122). That is, the community gives the concrete meaning to the otherwise abstract demands of the virtue of justice. Yet, since a virtue of justice is ordered toward normative standards of respect for all members of the human community, this prevents political virtue from collapsing into meaninglessness or providing legitimacy for bad states of affairs. Justice is responsive to the particular normative context while holding itself to an objective standard of right, which enables justice to present an ideal of betterment while still attending to the real demand that humans be treated with a baseline of respect and care. Discrete wrongs can thus be made right against the ideal of political justice, and long-term ills can be gradually addressed by the vision of a more just society.

To illustrate, the virtue of justice evaluates the nature of the relationships that exist, and are enabled, within a society. This is important, because in order to assess whether the institutions in a society express the virtue of justice, we must look at the ways in which they structure relations between people, and between groups. Are the relations fair? Are they respectful? This is clearly not solely a distributive question, and I will say more about that in a moment. Rather, when we ask if the relations between groups in a society exhibit justice, we are also asking whether they are characterised by equal regard and respect, or whether they are characterised by domination, subjugation, contempt, oppression and other markers of disrespect. Through various points in history, different groups experienced forms of domination, subjugation, or oppression. For example, the routine practice of institutionalising people with intellectual disabilities, forcing people to the margins of our society, expresses contempt and subjugation of these people (Rembis et al. 2018). While some with intellectual disability may not reach the capacity to enact a full set of rights and liberties, they are yet due the baseline respect that all members of the human community are owed. Political justice identifies that the relations between people with intellectual disabilities, people without, and major social institutions have gone wrong many times in the past, and points us toward a better, more respectful and inclusive way of life (Steele et al. 2023). So, political justice is expressed by an institution like public health when its structures – policies, campaigns, agendas, and priorities – express a commitment to normative standards of respect for all members of society and relations of non-domination, non-subjugation, and non-oppression between groups.

This interpretation of political virtue captures the ideas from both Aristotle and Aquinas that justice is an expression of a commitment to the good of society on behalf of agents who are making decisions for others, that is, agents with institutional power. A disposition amongst those agents who hold power to care for the good of society is revealed by attention to justice, “especially as expressed through rational and appropriate laws,” that is, through well-crafted structures (Porter 2016, p. 3). Aristotle posited that there is a link between rational structures and the development of a just, and flourishing, society. ‘Rational’ here means that the laws form a consistent set of structures aimed at achieving the proper end of human society. For Aristotle, this end was *eudaimonia*, often translated as ‘flourishing,’ but other ideas of the common good for society, or the liberal pluralist view that the end of laws is to arrange society so that many competing private ideas of the good can be achieved, are compatible with this way of conceptualising the ‘end’ of a system of laws. A system of laws must serve the interests of the people living under them in a way that conduces to the good. However, importantly for Aristotle’s ethics (as well as Kant’s and, to a certain extent, Rawls’), the end of human society is closely connected to the kind of being that we are: rational, political animals (Striker 2006). So, what it means to create a flourishing society is bound by what it means for rational animals to live well together. Whatever else the society does, it must ensure that people are treated with the basic respect due to a rational being, and must ensure the kinds of systems are in place to allow people to develop their capacities.

This means that oppressive laws are not rational. This might sound counter-intuitive insofar as some laws have been created specifically to exclude and subjugate some groups (like ‘Jim Crow’ laws in the 1890–1960 s United States), and are therefore goal-directed.^1^ However, that these laws have been purposely put in place by lawmakers to achieve a certain outcome does not make them rational laws. Though oppressive laws may be effective at excluding certain groups from obtaining various goods that they would otherwise be able to, or entitled to, obtain, oppressive laws are irrational precisely because they are unhinged from their proper object, insofar as they fail to treat human beings with the basic respect due to each member of humanity. So, the important point here is that the rationality of a system of laws is not judged based on whether or not some power-holding group has determined that these laws will effectively serve their own ends than another set of laws, but rather it is judged on whether a system of laws is properly justified in relation to the baseline conditions of respect for all members of society.

Oppression is incompatible with basic respect because it holds people down based on arbitrary features of groups, such as socio-economic status, skin colour, gender, or postcode. It takes a variety of forms, including marginalisation, powerlessness, exploitation, violence, cultural imperialism (Young 1990), testimonial injustice (Fricker 2007), stigmatisation (Goffman 2009), indifference (Geras 1998), and contempt (Hill 2000). For example, the oppressive force of poverty, in wealthy countries such as Australia, Canada, and the US, is the result of irrational systems of education, health, housing, financial, and market structures, which obstruct people from low-income backgrounds from achieving what they might were structures better organised. Homelessness is increasing in these nations, and this, too, is the result of irrational financial, housing, and market structures that could be modified by institutions. Though oppression happens mostly invisibly at the individual level, it is extraordinarily predictable and easily measurable at the population level. The oppression of poverty and homelessness is, in these rich countries, an indefensible and irrational political choice.

Considering justice as ordered to an objective standard of right, provided by the kinds of beings that we are has some particular strengths. It allows us to clearly see the mission of justice against oppression, domination, and subjugation. The mission to achieve justice by removing or revising irrational structures that arbitrarily hold people down is amenable to a wide range of political and religious worldviews. It further focuses attention on the ways in which institutions like public health, housing, or finance go about creating the conditions for (or against) justice by the structures they create and maintain. It also draws attention to the justice-oriented goals of institutional action, which in the case of public health must be more than increasing health equity. Non-oppression and non-subjugation denote relationships between groups that are marked by mutual respect and regard, and not by indifference or contempt. And finally, as briefly noted above, it is a principle of pluralism in liberal democracy that the government not get too involved in people’s lives, but that it create the background conditions in which people may pursue their personal ends. Non-oppression is consistent with, if not integral to, such an agenda.

In public health ethics and in political philosophy, the idea that justice is importantly connected to institutions and structures, and that these institutions and structures have clear and important impacts on people’s lives, is well established. Indeed, phenomena like oppression are most clearly seen in the patterns of health and illness, or suffering and flourishing, in a society rather than by looking at individual instances of illness and health. Further, these patterns have been linked to structures of advantage or disadvantage through the social determinants of health (Marmot and Allen 2014). So, in a society where there are significant structures of oppression already at work, a liberal commitment demands that institutions such as public health seek out these structures and remove them, insofar as they constitute arbitrary and irrational barriers.

I will return to this account in a moment, but first, I will explain why the usual distributive understanding of justice is not sufficient as an end of public health.

## Why not standard distributive justice?

When the question is about improving or achieving social justice, a common answer is that we must improve the distribution of the goods (and costs) of collective life amongst the members of society. This sense of justice describes the result of a distribution procedure, and there could be many such procedures that result in a just distribution. The most influential ideal liberal account of a procedure of distributive justice is, of course, John Rawls’s *Theory of Justice* (Rawls 1971). Rawls argues that his two principles of justice, which are: (1) equal basic liberties and (2) fair equality of opportunity + the difference principle, are the most reasonable and therefore most likely to be chosen out of a set list of options when those deciding how to set up society are placed in a position of radical uncertainty (a thought experiment known as the Original Position). According to Rawls, faced with ignorance about one’s social identity and standing, the rational chooser would opt for the set of principles that would minimize the badness of the worst-off social position relative to the best-off position. So, under conditions of uncertainty, the two principles of justice would be selected to establish basic social institutions and the way that they distribute benefits and burdens, including positions of responsibility (and power) within society.

Rawls’ theory has had its fair share of criticisms and support. Rather than rehearse the arguments for the two principles of justice, I want to focus on a quirk of this theory that I have always found interesting. Rawls considered the Original Position to be a highly uncertain place.^2^ Information is scarce, and stakes are high. Under such conditions, Rawls thought that each person’s self-interested aversion to risk would provide the right disposition for them to choose justice. Each would consider what would be in their own best interests (and the interest of their continuing genetic line, as the head of a household), should they end up in the worst possible social position. Facing uncertain outcomes, Rawls argued that people would choose to secure as much as they could for themselves via a set of basic liberties, and to make the worst possible social position as good as it could be – only slightly worse than the best possible social position, ideally. So, people would accept inequality between best and worst social positions only as a result of advantages that accrue to holding offices that are held open to all. Justice therefore becomes an output of mechanisms of self-interest, and Rawls thought this was a good feature of his theory. The two principles of justice were the best ones, he thought, because basing our social institutions upon self-interest would harness it in order to bring about good results. If we build self-interest into the mechanism of justice and make it seemingly to everyone’s advantage that the two principles are chosen, then it is less likely, by hypothesis, that we will end up in a situation of total competition, free-ridership, nepotism, and corruption.

As good as that sounds, I have frequently detected a problem lurking here. Principles of justice that are established on the basis of self-interest risk reinforcing the conditions under which self-interest is dominant (and this is, I believe, what has happened in society). Rawls sought to side-step a problem of moral motivation by establishing the two principles upon one of our common psychological traits. The problem he wanted to avoid is that even when we know what the right thing is to do, we may lack motivation to do it. Knowing what is right is not (always) sufficient to make us (want to) do what is right. This problem was noticed by Kant and by Mill, who respectively noted that the only thing that could make a person do what is right once they know what is right was having a good will or virtuous character.

So, side-stepping motivational issues plunges us into a different moral quagmire. Considering the role that structures play in shaping social conditions – as well as people’s characters, to some degree – setting up a system of distribution that is based on self-interest replicates and strengthens the motivations for self-interest, and this could have undesirable side-effects (including those, like free-riders, nepotism, or corruption, that Rawls hoped to avoid). Even if self-interest is a common psychological trait and a powerful motivation for many people, that does not mean it is the right basis for our institutions. We cannot depend on this as a motivation that will reliably result in just outcomes. And, basing social institutions upon self-interest provides us with no reasons to develop better characters, and instead it rewards individualism and self-interested thinking.

We see this in our social institutions now; muscular and institutionalised individualism erodes the cooperative basis of social justice, encouraging self-interested thinking, rewarding group-based division and competition, and undermining civic friendship. Rather than developing a disposition to work together and care for other groups in our wider community, we foster and rely on a disposition toward isolation from others. The ideal of justice as proposed by Rawls’ two principles is therefore not helping us to see what it would mean to be better or live in a more just society.^3^ Relying on one of our least virtuous traits to establish justice does not encourage moral development, and perhaps worse, it encourages the maintenance of current unjust structures.

Clearly, then, Rawlsian accounts of distributive justice in political philosophy and bioethics are unsuitable, maybe in general, but certainly for a virtue account. In addition to the problem of having self-interest at the core, such accounts tend to miss important justice conditions that take the various forms of oppression. For example, distributive accounts struggle to capture non-material aspects of justice, such as stigmatisation and expressions of gender- and race-based subordination, because they “cannot identify the gendered [and racialized] nature of the social norms that influence the distribution of the social basis of respect” (Fourie 2022, p. 33). Distributive accounts falter at taking account of the effects of embodiment and discrimination based on embodied life in a diverse society (Russell 2022, p. 198). Such accounts can also be ahistorical, either by setting aside the history that led to real injustice or imagining a fresh society without a history, to start from theoretical scratch (Fourie 2022, p. 30). And finally, distributive accounts struggle to capture the injustice of indifference or contempt, the “angry speech which reviles another [or] quiet, insidious speech which spreads calumny… deriding another by casting scorn upon him or her” (Aquinas 1516; Q72-76).

Thinking of justice as a function of principles of distribution, then, is not satisfactory in terms of capturing what we must do, and what our institutions must do, for us to live in a just society. I will now turn back to public health specifically, and the topic of health equity.

## Public health, health equity, and virtue

Perhaps the first thing to note is that public health aims at justice already, in the form of attempting to achieve greater health equity. Health equity is an outcome of distributions, at least according to the popular accounts of it provided in public health ethics (Powers and Faden 2006; de-Shalit and Wolff 2007; Daniels 2008). So, while justice is not foreign to public health, a virtue-orientation is still a significant change.

While thinking about justice as a virtue in institutions is under-theorised, it’s not completely new. Elizabeth Anderson and a few others have explored the aspects of justice that are related to virtue rather than functions of distributions. On the topic of testimonial injustice, for example, Anderson writes that “when the members of an organization jointly commit themselves to operating according to institutionalized principles that are designed to achieve testimonial justice, such as giving hearers enough time to make unbiased assessments, this is what it is for the organization itself to be testimonially just” (Anderson 2012, 168–169). To call an institution testimonially just means to evaluate its structures and practices as expressing virtue. And if we assign a virtue in this way, then we’re noticing that the institution is expressing a commitment to goodness in the form of justice, via the various operating procedures that will be designed in line with this commitment.

Pursuing the virtue of justice in public health means modifying public health’s deliberative practice so that justice is not only a distributive outcome of public health activity, but a good-making feature internal to public health structures. Virtue involves the motivations and practical reasoning of an actor, as much as it involves the means and the ends of an act. If we reflect on the social conditions that promote equity, it is clear that an orientation toward respect for all groups is a baseline requirement for virtue in public health. It is important that public health is motivated to achieve the virtue of justice while pursuing improvements in health. It must seek to achieve its health-related ends in a way that expresses a commitment to goodness by empowering and not oppressing (or contributing to the oppression of) different groups. This is what it means for excellent work in public health to be oriented toward justice as its telos; in all of the visible and invisible ways it structures society, to be acting excellently means public health is motivated by basic respect for all, and promoting and enabling just relations.

One might wonder what this means in practice and how we might see changes to what public health does. Incorporating virtue ethics into the practice of public health would mean that practical wisdom would guide deliberations about selecting noble ends and the finest means by which to achieve those ends. This would represent an important internal change, the outward effects of which would vary by practice and by context. For example, in pursuing the noble end of improving the health of the community, something like disease surveillance that happens mostly behind-the-scenes might be selected as the finest means, and since this is mostly an invisible process there may not be much outward change. Internally, however, there would be a modified set of reasons and justifications for the surveillance of populations, the ways in which populations were surveilled, and perhaps a change to the selection of communities to monitor. In other cases, like health promotion campaigns, which are primarily outwardly-directed and engage populations directly, we might expect a significant change in the ways in which these are carried out, or perhaps an abandonment of these means if practical wisdom shows that they are not fit for purpose.

Consider a real public health campaign about obesity in a population. The campaign featured an image of three stacked doughnuts on one side, with facts about overweight and obesity on the other side. The facts included that 1 in 4 children in this population was overweight or obese, that 34% of the current adult population was overweight or obese, and so on. The facts were connected to the stack of doughnuts by a series of lines. Despite the significant amount of evidence that correlated overweight and obesity with low socio-economic status and workplace conditions in the target population, the campaign correlated overweight and obesity with doughnuts, which were presumably used as a visual representation of unhealthy eating choices.

Scholars in bioethics have debated the ethics of fear campaigns and messages that stigmatise individual behaviours as a part of changing social attitudes toward health-related behaviour (Callahan 2013; Bayer 2008; Bayer and Fairchild 2016). Scholars have also debated whether such messages fulfil public health’s obligations to the public (Carter 2014, 2017); whether they respect individual liberty and autonomy (MacKay 2017); and simply whether they work or not – that is, do they change people’s choices, and lead to better health outcomes, understood, in this case, as lower rates of obesity. This scholarship notes that initiatives like health promotion campaigns commit a kind of cultural imperialism, forcing the values of the dominant, Anglo-Western middle class upon the other groups in society, without sufficient sensitivity to their values or their constraints (Abu-Odeh 2014). Campaigns such as the one described above both deploy and contribute to the stigma that fat people face, and also stigmatise a kind of food as aproxy for unhealthiness. The evidence for the effectiveness of such campaigns as means to achieve the ends of health promotion is shaky and easily confounded by other variables in the environment (such as tax changes, or restrictions on access). However, they are relatively cheap to produce, so governments can justify them based on their utility, even if they are not very effective. Virtue ethics, however, would not approve such means. This is first because practical wisdom would indicate that these means are not the finest by which we might achieve the goal of promoting health. Secondly, and relatedly, these campaigns are incompatible with justice; they are disrespectful, undermining, and contribute to the pattern of irrational structures that hold people down. We have evidence that oppressive structures inhibit people who are fat, and others as well, by blocking their potential to achieve goals and limiting their range of actions. This limits people from doing what they want to do. Groups who are stigmatised or face social exclusion will suffer in at least two ways: materially, by being blocked from achieving certain goods that they otherwise might obtain, and psychically, insofar as oppression undermines a person’s confidence in their own action model (Marmot and Allen 2014; Bland 2023; MacKay 2017; Abu-Odeh 2014). It is plausible that oppressive public health structures can make people sick.

For the institution of public health to capitalise on stigmatising associations and to create or maintain structures that disrespect people and hold them down is unacceptable. Doing so contributes to viciousness in the form of oppression, and undermines virtues – particularly of justice – while leaving root causes, like socio-economic status and workplace conditions unaddressed. This isn’t only the case for non-infectious disease; though I don’t have time to present it, a similar argument can be made about how COVID-19 lockdowns happened in some places and the impacts of those.

## Conclusion

As noted at the outset of this paper, walking the line between delivering public health outcomes while maintaining respect for groups and securing justice requires practical wisdom and careful deliberation. Virtue ethics is particularly well-suited to this. One likely result of incorporating virtue ethics into public health ethics and practice would be an improvement of decision-making procedures, and a clarification of what it means for public health to express a commitment to justice. In order for public health’s work to be defensible, the means by which it achieves its desired outcomes must be virtuous and be contrary to vice. Whether we consider campaigns, lockdowns, or disease surveillance, the health-regarding work of public health must be done for the sake of a healthier and more just society, and contrary to those forces of oppression that undermine justice and the concrete wellbeing of groups. Insofar as public health made its decisions with justice as non-oppression motivating its actions and serving as its telos, its activities would, I suggest, be more acceptable to a diverse society and more respectful to the plurality of values and life plans that can be legitimately pursued within it.

## Footnotes

1 With thanks to Steven Bland for pressing me on this.

2 Rawls discusses the degree of risk and risk aversion of reasoners in the Original Position in a couple of places (1971, pp. 118–119; 143–144) while also saying that people behind the Veil of Ignorance have no access to likelihoods or probabilities (p.134), creating a scenario in which reasoners have to make a choice under uncertainty.

3 Granted, most people outside of academia have not heard of Rawls. However, his *Theory of Justice* was very influential with politicians and political scientists when it was published (including Bill Clinton and the UK Labour Party), and has remained so.
